# BAC-BROWSER: The Tool for Visualization and Analysis of Prokaryotic Genomes

**DOI:** 10.3389/fmicb.2018.02827

**Published:** 2018-11-21

**Authors:** Irina A. Garanina, Gleb Y. Fisunov, Vadim M. Govorun

**Affiliations:** ^1^Federal Research and Clinical Centre of Physical-Chemical Medicine, Moscow, Russia; ^2^Shemyakin-Ovchinnikov Institute of Bioorganic Chemistry, Russian Academy of Sciences, Moscow, Russia; ^3^Moscow Institute of Physics and Technology, Dolgoprudny, Russia

**Keywords:** genome browser, viewer, visualization, genome, genes, bacteria, prokaryotes

## Abstract

Prokaryotes are actively studied objects in the scope of genomic regulation. Microbiologists need special tools for complex analysis of data to study and identification of regulatory mechanism in bacteria and archaea.

We developed a tool BAC-BROWSER, specifically for visualization and analysis of small prokaryotic genomes. BAC-BROWSER provides tools for different types of analysis to study a wide set of regulatory mechanisms of prokaryotes:

- transcriptional regulation by transcription factors (TFs), analysis of TFs, their targets, and binding sites.
- other regulatory motifs, promoters, terminators and ribosome binding sites
- transcriptional regulation by variation of operon structure, alternative starts or ends of transcription.
- non-coding RNAs, antisense RNAs
- RNA secondary structure, riboswitches
- GC content, GC skew, codon usage

BAC-browser incorporated free programs accelerating the verification of obtained results: primer design and oligocalculator, vector visualization, the tool for synthetic gene construction. The program is designed for Windows operating system and freely available for download in http://smdb.rcpcm.org/tools/index.html.

## Introduction

Prokaryotes are the great source for discovery of new regulatory mechanisms. Their diversity and relative simplicity of genome organization make them suitable objects for different -omics and comparative analysis. Regulation by transcription factors is considered as well described for bacteria, but even for model bacteria *Escherichia coli* and *Bacillus subtilis* new TFs and targets are still being identified (Keseler et al., 2013; Gama-Castro et al., 2016; Fang et al., 2017; Belliveau et al., 2018; Gao et al., 2018). Since the low conservation of regulatory networks, regulation of other bacteria, including important pathogens, remains obscure (Lozada-Chávez et al., 2006; Rodionov, 2007; Fisunov et al., 2016; Eckweiler et al., 2018). Operon structure is one of the interesting and poorly studied features of prokaryotes. An operon is defined as a set of genes transcribed en bloc, so operon consists of several genes transcribed and regulated together (Jacob et al., 1960). New data about bacterial transcription showed that operon structure is not stable and can vary in different conditions (Koide et al., 2009; Mao et al., 2015; Junier and Rivoire, 2016). To cope with this discrepancy, genes transcribed and regulated together were called transcriptional units (TU) (Cho et al., 2009; Junier and Rivoire, 2016) and now term operon has more meaning in terms of functional rather than direct transcriptional link between genes (Okuda et al., 2007). Identification of TUs is a new task that has to be resolved to understand transcriptional regulation. Exact mapping of TUs can facilitate identification of new riboswitches, non-coding RNA, antisense transcripts (Sharma et al., 2010; Boutard et al., 2016). The same TU may have multiple TSSs (transcription start sites) and transcription ends. Alternative TSSs in bacteria are found for 15–60% genes and operons (Stazic and Voß, 2016), they are also involved in transcription regulation (Cho et al., 2009; Li et al., 2015). To characterize both poorly studied regulatory mechanisms and classical ways of regulation is needed to use complex information about transcription and translation and combine it with genomics data. For this purpose were developed special tools and programs (Pavlopoulos et al., 2015).

There are many specific tools for TU mapping, TSS and terminator prediction. They use various strategies for data analysis (Amman et al., 2014; Fortino et al., 2014, 2016; Čuklina et al., 2016; Promworn et al., 2017), and some can visualize the results (McClure et al., 2013; Hilker et al., 2016). However, these tools generally concentrated on one or few aspects of regulation. Wide set of tools utilize known algorithms for high-throughput data analysis and genomes visualization but none of them allow analysis of complex operon structure of prokaryotes (Carver et al., 2012; Okonechnikov et al., 2012; Lechat et al., 2013; Dietrich et al., 2014).

We developed new genome browser BAC-BROWSER designed for molecular biologists and microbiologists that combine user-friendly interface with multiple functions for prokaryotic regulation analysis. Using RNA-seq read coverage BAC-BROWSER can identify TSSs and terminators, map and visualize TUs. BAC-BROWSER provides a wide set of algorithms for analysis of genes, proteins, regulatory motifs and structural elements, like hairpins and riboswitches. Furthermore, the program facilitates subsequent verification of computational results with built-in tools for molecular biology analysis. BAC-BROWSER includes free modules for primer design, vector representation, and analysis. Based on BAC-BROWSER prediction we identified TSSs and gene promoters that helped to reveal a new mechanism of transcriptional regulation and reconstruct transcriptional control network for three bacteria species (Mazin et al., 2014; Fisunov et al., 2016).

## Implementation

### General Information

BAC-BROWSER software was written in VB.NET 9.0. The program loads genomes in Fasta, Genbank, and GFF format.

### Analysis of Coverage and Transcription Unit Mapping

BAC-BROWSER intakes coverage data in SAM/BAM format or in own format. BAC-BROWSER format represents the array of values, where each one represents coverage at a given position starting from the first nucleotide. The SAM format can be converted into BAC-BROWSER format. The coverage dataset can be assembled into a single table file, which simplifies loading of data. The coverage can be represented as full read coverage or first nucleotide coverage. In the second method only first nucleotide contributes to the coverage, while the remaining read is skipped. This representation is used for analysis of 5′-end enriched RNA-seq coverage (Mazin et al., 2014).

The algorithm of transcription unit identification from the coverage have been described in Mazin et al. (2014). Briefly, the algorithm calculates coverage derivative and identifies its local extremes, which correspond to local steps in coverage. The steps may originate from TSSs, transcriptional terminators and RNA ends produced by RNA processing as well as coverage noise produced fortuitously. The algorithm further splits coverage into intervals between consecutive steps and identifies statistical significance of coverage difference between adjacent intervals. In current implementation we use standard deviation, which threshold can be manually set. The original implementation described a quasi-Poisson distribution test. Then if the difference is statistically insignificant the step is removed and intervals are joined. The process repeats iteratively until convergence. The final set of intervals can be assigned as transcription units (operons). The borders between the intervals correspond to TSSs and transcriptional terminators, respectively (Mazin et al., 2014). So, this algorithm identifies TSSs, terminators, and TUs. However, its accuracy depends on the quality of data and parameters the user chooses.

The exact identification of TSSs with single-nucleotide resolution requires first nucleotide coverage data from 5′-end enriched RNA-seq library. The method is different from the algorithm for TU identification and also have been published (Mazin et al., 2014). The program scans the coverage for local maxima, which correspond to TSSs. The algorithm accounts for background signal to identify maxima that are above the noise threshold. The latter is dynamically calculated for the local sequence area (parameters are user-adjusted).

It is also possible to export normalized coverage (FPKM) of annotated features for further analysis. A coverage of a single gene is normalized to the total library coverage or to the coverage of CDSs (Anders and Huber, 2010). The latter excludes overrepresented features like rRNAs and tRNAs that may introduce quantitative bias.

### Applications for Sequence Analysis, Oligonucleotide Thermodynamics and Secondary Structure Analysis and Motif Search

Standard algorithms for sequence search are implemented in the application (Altschul et al., 1990). At first stage query and subject sequences are splitted into K-words. K-words are indexed and the matrix of matched hits is built. Matched hits serve as seeds to expand alignment. The gaps between seeds are filled by Wunch-Needleman algorithm (Needleman and Wunsch, 1970). A user can adjust thresholds for sequence similarity, minimal alignment length and length of seeds.

Positional weight matrixes (PWMs) are built from a user-supplied set of aligned sequences. For logo plot construction user can apply log-likelihood score or Shannon positional entropy (Li and Tompa, 2006). PWMs are stored in text format and can be easily edited. External or manually constructed PWMs are suitable for search. The program scans a genome and finds PWM matches above the threshold score manually selected by a user. The score is calculated by the standard method as a sum of frequencies of matched nucleotides. For motif *de novo* search we implemented modified MEME algorithm (Bailey and Elkan, 1994).

Thermodynamic parameters for oligonucleotides are calculated by the modified Allawi and SantaLucia’s the nearest-neighbor method for DNA (Allawi and SantaLucia, 1997). Thermodynamic parameters for RNA hairpins (used in hairpin finder at genomic view window) are calculated by the nearest-neighbor algorithm for RNA (SantaLucia, 1998). Secondary structures and of oligonucleotide dimers are calculated using iterative annealing with subsequent calculation of duplex stability. For calculation of hairpins, the oligonucleotide is split into two halves and iterative annealing of halves is performed. A search of secondary structures in a genome is performed in a sliding window. For each subsequence the algorithm calculates the hairpin stability in the same way as for oligonucleotide (see above), but with RNA thermodynamic parameters.

The identification of repetitive elements is performed in a sliding window. For each subsequence the algorithm identifies if N nucleotides separated by the spacer of K length form direct or inverted repeats.

### Tools for Molecular Biology

PCR primer design in the program includes the following methods: automatic primer design for the given sequence and manual primer design. The first method designs a list of primers that correspond to the manual set of parameters like Tm, amplicon length and secondary structure stability. The algorithm makes the list of subsequences from the sequence to which the primers have to be designed. Then the algorithm filters subsequences until the appropriate ones are found. The second method allows designing oligonucleotide directly on DNA sequence within the viewer window. A user can move and resize oligonucleotide on genomic sequence and Tm is calculated on-flight. This method can be used to design oligonucleotides for gene synthesis or probes for real-time PCR. Tm is calculated by the modified Allawi and SantaLucia’s thermodynamics method (Allawi and SantaLucia, 1997). Calculation of oligonucleotide concentration from OD is performed by approximation of Beer-Lambert law by the following equation: OD / (nA × 15200 + nG × 12010 + nC × 7050 + nT × 8400 + M), where nA, nG, nC, and nT is the number of respective nucleotides and M corresponds for absorbance of DNA modifications (if present).

The protein isoelectric point is calculated based on the Henderson-Hasselbach equation and table values of pK for charged amino acids. The program iteratively adjusts pH until the isoelectric point is reached.

## Results and Discussion

### Sequence and Coverage Analysis

In BAC-BROWSER there are available linear and circular sequence representations. An easy and usable interface enables to manipulate with annotation, gene sequences, mark and retrieve nucleotide fragments or directly analyze them. BAC-BROWSER allows manual correction, addition, and import of annotation. BAC-BROWSER provides simple extraction of a single or multiple genes or protein sequences. The program can upload files with more than one sequence and show it as one genome. Sequence search is available in the application. BAC-BROWSER performs a search of nucleotide and protein sequences with the customizable percent of identity and can do the simultaneous search of several sequences uploaded in Fasta file. The program readily works with short sequences as vectors, viral and bacterial genomes. BAC-browser will work with the human genome and other large genomes, but it is not designed for this and the analysis will take a long time.

**FIGURE 1:**
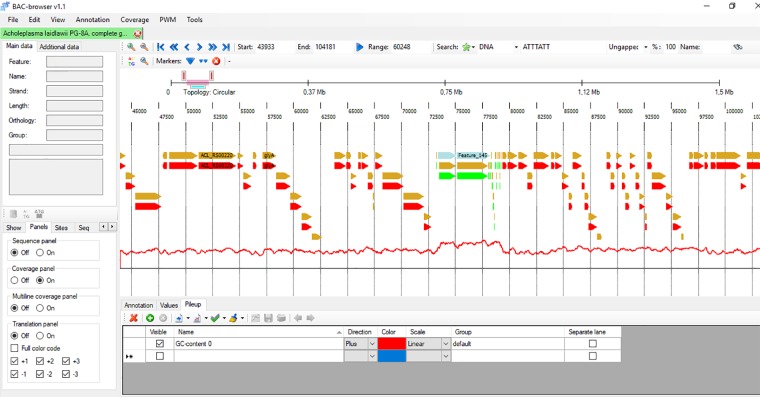
The screenshot of BAC-browser. In genome panel the bacterium *Acholeplasma laidlawii* genome is shown. You can see genes as arrows, the red line under genes shows GC content calculated in 200 nt window. On this example the local GC content increase is observed in the region of 73000–78000 nts there ribosomal RNAs are located.

In BAC-BROWSER, there are more than 15 tools implemented for sequence and associated coverage analysis:

- genome sequence searching and editing;
- tools for editing genome annotation: annotation amendment, importing and exporting of features, open reading frame (ORF) prediction;
- tools for motif search and analysis like PWM scanning and building, and also *de novo* motif search;
- tools for repeat and hairpin formation search;
- restriction site search and enzyme mapping;
- BLAST search;
- GC content (Figure 1) and GC skew calculators, and codon usage.

RNA-seq analysis output in SAM format can be directly loaded by the application. SAM/BAM file user can obtain from programs for RNA-seq analysis such as Bowtie, BWA or TopHat (Li and Durbin, 2009; Langmead and Salzberg, 2012; Kim et al., 2013). Read alignments are automatically converted into nucleotide coverage. BAC-BROWSER can perform coverage normalization and log transformation. A user chooses the type of coverage representation depending on RNA-seq library type. BAC-BROWSER generates standard nucleotide coverage and coverage obtained with first nucleotides of reads. The second type of coverage we recommend to use for 5′ enriched RNA-seq (Sharma et al., 2010; Creecy and Conway, 2015). This type of RNA-seq library preparation used for exact TSSs mapping and quantitate transcript analysis (Mazin et al., 2014; Fisunov et al., 2016). The program also can load other quantitate or qualitative data in simple and universal BAC-BROWSER format. It can be used for methylation, SNP or GC content display. So, BAC-BROWSER provides a simultaneous view on biological data of different types and keeps it in lightweight universal format.

### Prediction of Transcriptional Units

In the program we implemented a method for condition-specific TU analysis in bacteria. The program analyses RNA-seq data for fast identification of TSSs, transcription terminators and TUs. This method and algorithm we used in our published work (Mazin et al., 2014), but here we want to compare it with another method and show accuracy on simulated data. For real data testing, we used previously published *E. coli* dataset (Conway et al., 2014). To get coverage were used processed RNA-seq data. Derived results correspond to results of Conway et al. (2014) and show high sensitivity (0.91) (Table 1).

**Table 1:** Comparison of the results of TU mapping.

	TSSs	Terminators	TUs
BAC-BROWSER	2038	2068	2321
Conway et al., 2014	2122	1774	2547

**FIGURE 2:**
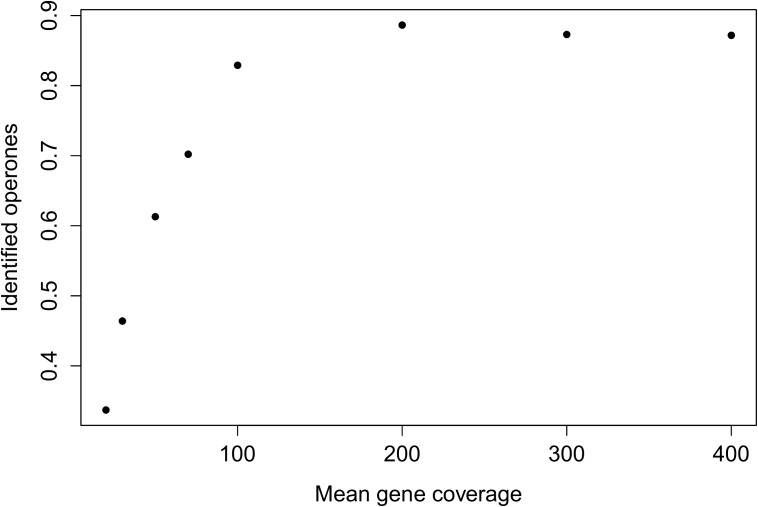
Dependence between mean gene coverage in simulated library and fraction of identified operons.

For read count simulation was used negative binomial distribution (Frazee et al., 2015). We simulated standard RNA-seq transcriptome reads varying mean coverage of gene from 20 to 400 reads per nucleotide, coverage for each transcript was drawn from the normal distribution. Results of the testing show, that implemented in BAC-BROWSER algorithm for TU identification is sensible for mean transcript coverage and can identify about 90% of TUs (Figure 2).

### Tools for Molecular Biology

BAC-BROWSER features a set of additional functions for molecular biology: oligonucleotide design, codon usage optimization for amino acid sequences and identification of restriction sites. Oligonucleotide design module includes automatic PCR primer design for a given sequence and manual oligonucleotide design. The program calculates oligonucleotide parameters including dG, Tm and secondary structure. Modifications of 5′ and 3′ ends like fluorophores and quenchers are accounted for. Embedded oligonucleotide calculator can calculate the desired dilutions from OD or molar concentration. Manual oligonucleotide design runs directly in genome view window and allows on-flight calculation of thermodynamic parameters. This tool can be used to design real-time PCR probes, cloning primers and synthetic sequences. The codon optimization module includes calculation of codon usage frequencies for the given genome and re-encoding of the particular amino acid sequence using given codon-usage table. BAC-BROWSER provides the module for *in silico* 2D electrophoresis analysis. Based on protein properties, the program constructs a theoretical 2D map for all genes in the analyzed genome.

## Conclusion

We developed an easy, fast and multifunctional application BAC-BROWSER for prokaryotic genome visualization and analysis. The program combines popular algorithms and methods for better interpretation and analysis of complex data and provides tools for subsequent verification of results with molecular biology methods. The program freely available and will be improved and supplemented in future versions which will be also available for the research community. In particular, we plan to make the web version of the program accessible to users with any operating system. Now BAC-browser has several limitations: it works only in Windows operating system and designed for analysis of small genomes up to 20 Mb in length. The manual describing file formats, usage of the program and parameters of tools is available in http://smdb.rcpcm.org/tools/index.html.

## Data Availability

Program in freely available in the SMDB website http://smdb.rcpcm.org/tools/index.html under the GNU GPL license. Operating systems: Windows XP and later versions. Programming language: VB.NET 9.0. Other requirements: none. Any restrictions to use by non-academics: none.

## Author Contributions

GF contributed to the software design, software testing, and the drafting of the manuscript. IG contributed to the software testing, bioinformatics analysis, data interpretation, and the drafting of the manuscript. VG contributed to the design of the study and the drafting of the manuscript.

## Conflict of Interest Statement

The authors declare that the research was conducted in the absence of any commercial or financial relationships that could be construed as a potential conflict of interest.

## References

[B1] Allawi H. T. SantaLucia J. (1997). Thermodynamics and NMR of internal G.T mismatches in DNA. *Biochemistry* 36 10581–10594. 10.1021/bi962590c 9265640

[B2] Altschul S. F. Gish W. Miller W. Myers E. W. Lipman D. J. (1990). Basic local alignment search tool. *J. Mol. Biol.* 215 403–410. 10.1016/S0022-2836(05)80360-2 2231712

[B3] Amman F. Wolfinger M. T. Lorenz R. Hofacker I. L. Stadler P. F. Findeiß S. (2014). TSSAR: TSS annotation regime for dRNA-seq data. *BMC Bioinformatics* 15:89. 10.1186/1471-2105-15-89 24674136 PMC4098767

[B4] Anders S. Huber W. (2010). Differential expression analysis for sequence count data. *Genome Biol.* 11:R106. 10.1186/gb-2010-11-10-r106 20979621 PMC3218662

[B5] Bailey T. L. Elkan C. (1994). Fitting a mixture model by expectation maximization to discover motifs in biopolymers. *Proc. Int. Conf. Intell. Syst. Mol. Biol.* 2 28–36. 7584402

[B6] Belliveau N. M. Barnes S. L. Ireland W. T. Jones D. L. Sweredoski M. J. Moradian A. (2018). Systematic approach for dissecting the molecular mechanisms of transcriptional regulation in bacteria. *Proc. Natl. Acad. Sci. U.S.A.* 115 E4796–E4805. 10.1073/pnas.1722055115 29728462 PMC6003448

[B7] Boutard M. Ettwiller L. Cerisy T. Alberti A. Labadie K. Salanoubat M. (2016). Global repositioning of transcription start sites in a plant-fermenting bacterium. *Nat. Commun.* 7:13783. 10.1038/ncomms13783 27982035 PMC5171806

[B8] Carver T. Harris S. R. Berriman M. Parkhill J. McQuillan J. A. (2012). Artemis: an integrated platform for visualization and analysis of high-throughput sequence-based experimental data. *Bioinformatics* 28 464–469. 10.1093/bioinformatics/btr703 22199388 PMC3278759

[B9] Cho B.-K. Zengler K. Qiu Y. Park Y. S. Knight E. M. Barrett C. L. (2009). The transcription unit architecture of the *Escherichia coli* genome. *Nat. Biotechnol.* 27 1043–1049. 10.1038/nbt.1582 19881496 PMC3832199

[B10] Conway T. Creecy J. P. Maddox S. M. Grissom J. E. Conkle T. L. Shadid T. M. (2014). Unprecedented high-resolution view of bacterial operon architecture revealed by RNA sequencing. *mBio* 5:e01442-14. 10.1128/mBio.01442-14 25006232 PMC4161252

[B11] Creecy J. P. Conway T. (2015). Quantitative bacterial transcriptomics with RNA-seq. *Curr. Opin. Microbiol.* 23 133–140. 10.1016/j.mib.2014.11.011 25483350 PMC4323862

[B12] Čuklina J. Hahn J. Imakaev M. Omasits U. Förstner K. U. Ljubimov N. (2016). Genome-wide transcription start site mapping of *Bradyrhizobium japonicum* grown free-living or in symbiosis - a rich resource to identify new transcripts, proteins and to study gene regulation. *BMC Genomics* 17:302. 10.1186/s12864-016-2602-9 27107716 PMC4842269

[B13] Dietrich S. Wiegand S. Liesegang H. (2014). TraV: a genome context sensitive transcriptome browser. *PLoS One* 9:e93677. 10.1371/journal.pone.0093677 24709941 PMC3977867

[B14] Eckweiler D. Dudek C.-A. Hartlich J. Brötje D. Jahn D. (2018). PRODORIC2: the bacterial gene regulation database in 2018. *Nucleic Acids Res.* 46 D320–D326. 10.1093/nar/gkx1091 29136200 PMC5753277

[B15] Fang X. Sastry A. Mih N. Kim D. Tan J. Yurkovich J. T. (2017). Global transcriptional regulatory network for *Escherichia coli* robustly connects gene expression to transcription factor activities. *Proc. Natl. Acad. Sci. U.S.A.* 114 10286–10291. 10.1073/pnas.1702581114 28874552 PMC5617254

[B16] Fisunov G. Y. Garanina I. A. Evsyutina D. V. Semashko T. A. Nikitina A. S. Govorun V. M. (2016). Reconstruction of transcription control networks in mollicutes by high-throughput identification of promoters. *Front. Microbiol.* 7:1977. 10.3389/fmicb.2016.01977 27999573 PMC5138195

[B17] Fortino V. Smolander O.-P. Auvinen P. Tagliaferri R. Greco D. (2014). Transcriptome dynamics-based operon prediction in prokaryotes. *BMC Bioinformatics* 15:145. 10.1186/1471-2105-15-145 24884724 PMC4235196

[B18] Fortino V. Tagliaferri R. Greco D. (2016). CONDOP: an R package for CONdition-dependent operon predictions. *Bioinformatics* 32 3199–3200. 10.1093/bioinformatics/btw330 27296981

[B19] Frazee A. C. Jaffe A. E. Langmead B. Leek J. T. (2015). Polyester: simulating RNA-seq datasets with differential transcript expression. *Bioinformatics* 31 2778–2784. 10.1093/bioinformatics/btv272 25926345 PMC4635655

[B20] Gama-Castro S. Salgado H. Santos-Zavaleta A. Ledezma-Tejeida D. Muñiz-Rascado L. García-Sotelo J. S. (2016). RegulonDB version 9.0: high-level integration of gene regulation, coexpression, motif clustering and beyond. *Nucleic Acids Res.* 44 D133–D143. 10.1093/nar/gkv1156 26527724 PMC4702833

[B21] Gao Y. Yurkovich J. T. Seo S. W. Kabimoldayev I. Drger A. Chen K. (2018). Systematic discovery of uncharacterized transcription factors in *Escherichia coli* K-12 MG1655. *Nucleic Acids Res.* 10.1093/nar/gky752 [Epub ahead of print]. 30137486 PMC6237786

[B22] Hilker R. Stadermann K. B. Schwengers O. Anisiforov E. Jaenicke S. Weisshaar B. (2016). ReadXplorer 2-detailed read mapping analysis and visualization from one single source. *Bioinformatics* 32 3702–3708. 10.1093/bioinformatics/btw541 27540267 PMC5167064

[B23] Jacob F. Perrin D. Sanchez C. Monod J. (1960). Operon: a group of genes with the expression coordinated by an operator. *C. R. Hebd. Seances Acad. Sci.* 250 1727–1729. 14406329

[B24] Junier I. Rivoire O. (2016). Conserved units of co-expression in bacterial genomes: an evolutionary insight into transcriptional regulation. *PLoS One* 11:e0155740. 10.1371/journal.pone.0155740 27195891 PMC4873041

[B25] Keseler I. M. Mackie A. Peralta-Gil M. Santos-Zavaleta A. Gama-Castro S. Bonavides-Martínez C. (2013). EcoCyc: fusing model organism databases with systems biology. *Nucleic Acids Res.* 41 D605–D612. 10.1093/nar/gks1027 23143106 PMC3531154

[B26] Kim D. Pertea G. Trapnell C. Pimentel H. Kelley R. Salzberg S. L. (2013). TopHat2: accurate alignment of transcriptomes in the presence of insertions, deletions and gene fusions. *Genome Biol.* 14:R36. 10.1186/gb-2013-14-4-r36 23618408 PMC4053844

[B27] Koide T. Reiss D. J. Bare J. C. Pang W. L. Facciotti M. T. Schmid A. K. (2009). Prevalence of transcription promoters within archaeal operons and coding sequences. *Mol. Syst. Biol.* 5:285. 10.1038/msb.2009.42 19536208 PMC2710873

[B28] Langmead B. Salzberg S. L. (2012). Fast gapped-read alignment with Bowtie 2. *Nat. Methods* 9 357–359. 10.1038/nmeth.1923 22388286 PMC3322381

[B29] Lechat P. Souche E. Moszer I. (2013). SynTView - an interactive multi-view genome browser for next-generation comparative microorganism genomics. *BMC Bioinformatics* 14:277. 10.1186/1471-2105-14-277 24053737 PMC3849071

[B30] Li H. Durbin R. (2009). Fast and accurate short read alignment with burrows-wheeler transform. *Bioinformatics* 5 1754–1760. 10.1093/bioinformatics/btp324 19451168 PMC2705234

[B31] Li J. Qi L. Guo Y. Yue L. Li Y. Ge W. (2015). Global mapping transcriptional start sites revealed both transcriptional and post-transcriptional regulation of cold adaptation in the methanogenic archaeon Methanolobus psychrophilus. *Sci. Rep.* 5:9209. 10.1038/srep09209 25784521 PMC5378194

[B32] Li N. Tompa M. (2006). Analysis of computational approaches for motif discovery. *Algorithms Mol. Biol.* 1:8. 10.1186/1748-7188-1-8 16722558 PMC1540429

[B33] Lozada-Chávez I. Janga S. C. Collado-Vides J. (2006). Bacterial regulatory networks are extremely flexible in evolution. *Nucleic Acids Res.* 34 3434–3445. 10.1093/nar/gkl423 16840530 PMC1524901

[B34] Mao X. Ma Q. Liu B. Chen X. Zhang H. Xu Y. (2015). Revisiting operons: an analysis of the landscape of transcriptional units in *E. coli*. *BMC Bioinformatics* 16:356. 10.1186/s12859-015-0805-8 26538447 PMC4634151

[B35] Mazin P. V. Fisunov G. Y. Gorbachev A. Y. Kapitskaya K. Y. Altukhov I. A. Semashko T. A. (2014). Transcriptome analysis reveals novel regulatory mechanisms in a genome-reduced bacterium. *Nucleic Acids Res.* 42 13254–13268. 10.1093/nar/gku976 25361977 PMC4245973

[B36] McClure R. Balasubramanian D. Sun Y. Bobrovskyy M. Sumby P. Genco C. A. (2013). Computational analysis of bacterial RNA-Seq data. *Nucleic Acids Res.* 41 e140. 10.1093/nar/gkt444 23716638 PMC3737546

[B37] Needleman S. B. Wunsch C. D. (1970). A general method applicable to the search for similarities in the amino acid sequence of two proteins. *J. Mol. Biol.* 48 443–453. 10.1016/0022-2836(70)90057-4 5420325

[B38] Okonechnikov K. Golosova O. Fursov M. Ugene team. (2012). Unipro UGENE: a unified bioinformatics toolkit. *Bioinformatics* 28 1166–1167. 10.1093/bioinformatics/bts091 22368248

[B39] Okuda S. Kawashima S. Kobayashi K. Ogasawara N. Kanehisa M. Goto S. (2007). Characterization of relationships between transcriptional units and operon structures in *Bacillus subtilis* and *Escherichia coli*. *BMC Genomics* 8:48. 10.1186/1471-2164-8-48 17298663 PMC1808063

[B40] Pavlopoulos G. A. Malliarakis D. Papanikolaou N. Theodosiou T. Enright A. J. Iliopoulos I. (2015). Visualizing genome and systems biology: technologies, tools, implementation techniques and trends, past, present and future. *Gigascience* 4:38. 10.1186/s13742-015-0077-2 26309733 PMC4548842

[B41] Promworn Y. Kaewprommal P. Shaw P. J. Intarapanich A. Tongsima S. Piriyapongsa J. (2017). ToNER: a tool for identifying nucleotide enrichment signals in feature-enriched RNA-seq data. *PLoS One* 12:e0178483. 10.1371/journal.pone.0178483 28542466 PMC5444824

[B42] Rodionov D. A. (2007). Comparative genomic reconstruction of transcriptional regulatory networks in bacteria. *Chem. Rev.* 107 3467–3497. 10.1021/cr068309 17636889 PMC2643304

[B43] SantaLucia J. (1998). A unified view of polymer, dumbbell, and oligonucleotide DNA nearest-neighbor thermodynamics. *Proc. Natl. Acad. Sci. U.S.A.* 95 1460–1465. 10.1073/pnas.95.4.1460 9465037 PMC19045

[B44] Sharma C. M. Hoffmann S. Darfeuille F. Reignier J. Findeiss S. Sittka A. (2010). The primary transcriptome of the major human pathogen *Helicobacter pylori*. *Nature* 464 250–255. 10.1038/nature08756 20164839

[B45] Stazic D. Voß B. (2016). The complexity of bacterial transcriptomes. *J. Biotechnol.* 232 69–78. 10.1016/j.jbiotec.2015.09.041 26450562

